# MGMT promoter methylation is a strong prognostic factor for survival after progression in high-grade gliomas

**DOI:** 10.1186/s41016-024-00375-2

**Published:** 2024-07-24

**Authors:** Jing Zhang, Xiaoguang Qiu, Jin Feng, Yanwei Liu

**Affiliations:** Department of Radiation Oncology, Beijing Tiantan Hospital, Capital Medical University, Beijing, 100070 China

**Keywords:** High-grade gliomas, MGMT promoter methylation, Survival after the first progression

## Abstract

**Background:**

High-grade gliomas (HGGs) have a rapid relapse and short survival. Studies have identified many clinical characteristics and biomarkers associated with progression-free survival (PFS) and over-survival (OS). However, there has not yet a comprehensive study on survival after the first progression (SAP).

**Methods:**

From CGGA and TCGA, 319 and 308 HGGs were confirmed as the first progression. The data on clinical characteristics and biomarkers were analyzed in accordance with OS, PFS, and SAP.

**Results:**

Analysis of 319 patients from CGGA, significant predictors of improved OS/PFS/SAP were WHO grade, MGMT promoter methylation, and Ki-67 expression in univariate analysis. Further multivariate analysis showed MGMT promoter methylation and Ki-67 expression were independent predictors. However, an analysis of 308 patients from TCGA found MGMT promoter methylation is the only prognostic marker. A longer SAP was observed in patients with methylated MGMT promoter after standard chemoradiotherapy. In our data, HGGs could be divided into low, intermediate, and high-risk groups for SAP by MGMT methylation and Ki-67 expression.

**Conclusions:**

Patients with MGMT promoter methylation have a prolonger SAP after standard chemoradiotherapy. HGGs could be divided into low, intermediate, and high-risk groups for SAP according to MGMT status and Ki-67 expression.

## Background

High-grade gliomas (HGGs) are the most lethal type of primary brain tumor and often demonstrate resistance to traditional cancer treatments, such as surgery, chemotherapy, and radiation. Maximal resection and postoperative concurrent chemoradiotherapy, followed by adjuvant chemotherapy are the standard treatment for patients with HGGs [1]. However, the OS and PFS were not significantly improved in the last decades, even though the temozolomide was shown to add a few months. The majority of patients with HGGs had relapse or progression in a short time, even during the treatment. The progressive tumor grows faster and shows resistance to chemoradiotherapy. The survival time of patients with progressive tumors is sharply shortened [2–5].

More reports have emerged in recent years on assessing survival outcomes and determining predictors of survival. OS and PFS are the most commonly used as endpoints in glioma studies. In recent years, mutated IDH1 and MGMT promoter methylation were the most common research targets in gliomas and they were associated with longer OS and PFS [6–9]. IDH mutation status at presentation was still found to be of prognostic significance, and MGMT promoter methylation was shown as an effective predictor to guide clinical TMZ in patients with HGGs [6]. Patients with MGMT promoter methylation received the standard schedule (TMZ at a daily dose of 75/m^2^ throughout the entire duration of RT followed by 6 cycles of adjuvant TMZ every 28 days according to the standard 5-day schedule) can benefit from 12 months OS to 14.6 months [10]. A randomized trial in patients with recurrent high-grade glioma demonstrated the independent prognostic significance of IDH1/2 and MGMT methylation status for prolonged OS [11, 12]. Other studies confirm that IDH1 and MGMT were associated with PFS in HGGs [13]. However, whether or not these markers have still prognostic or predictive value for survival after the first progression (SAP) has not been systematically discussed.

The aims of this study were to assess whether mutated IDH1 and MGMT promoter methylation which were associated with OS and PFS have prognostic or predictive value for SAP and contribute to the prognostic classification of SAP. We collected 319 HGGs which were confirmed the first recurrent or progression by MRI or CT after maximum resection followed by radiotherapy and/or temozolomide chemotherapy. The application of Cox regression analysis identifies the associated factors with the OS/PFS/SAP. The results were validated by 308 HGGs from TCGA. This study has the potential to accurate assessment of SAP prognostic groups in patients with HGGs and to influence clinical decision-making.

## Methods

### Patients data

The clinical data of the patients are from The Chinese Glioma Genome Atlas (CGGA, http://www.cgga.org.cn) and The Cancer Genome Atlas (TCGA, http://cancergenome.nih.gov). The clinical data include some classical markers that were used to analyze the prognostic factor for the survival of the patients. We only chose the patients: who underwent surgical resection and whose pathology diagnosis was definitely diagnosed by 2 neuropathologists according to the guidelines of the 2007 WHO classification; who was confirmed recurrent or progression; and who conducted resection followed by radiotherapy or TMZ chemotherapy. The informed consent was obtained from each of the patients and the institutional review boards approved the research.

### Statistical analysis

We used GraphPad Prism 5.0 statistical software (LaJolla, CA, USA) and SPSS 16.0 (Armonk, NY, USA) to do the statistical analysis. Univariate and multivariate Cox regression analysis was done by SPSS16.0, including age, gender, TCGA subtype, MGMT methylation status, IDH1 mutation status, TP53 mutation status, EGFR Amplification status, Ki-67 expression, 1p/19q status, involved lobe, extent of resection, operation or TMZ after progression. A two-sided *P* value < 0.05 was regarded as significant. We use GraphPad Prism 5.0 to do Kaplan–Meier survival analysis to estimate the survival distributions by the cut points at the median. The log-rank test was used to assess the statistical significance by the *p* value < 0.05.

### Molecular evaluation

Tumor tissue samples were put into liquid nitrogen after the surgery until the next use. Genomic DNA was extracted by using the QIAamp DNA Mini Kit (Qiagen). The IDH1/2 mutation status was analyzed by DNA pyrosequencing. The MGMT promoter methylation status was analyzed by DNA pyrosequencing. The 1p/19q co-deletion was detected by fluorescence in situ hybridization. The EGFR amplification and Ki-67 expression were detected by immunohistochemistry. Anti-ki-67 at a dilution of 1:100. The staining intensity was jointly scored by two pathologists without knowledge of clinical information on a scale of 0–3 (0, negative; 1, slight positive; 2, moderate positive; 3, intense positive). And scale of 0 and 1 and a scale of 2 and 3 indicated low and high expression of the above proteins, respectively. Controls without primary antibodies and positive control tissues were included in all experiments to ensure the quality of staining.

## Results

### Patients and survival

**Fig. 1:**
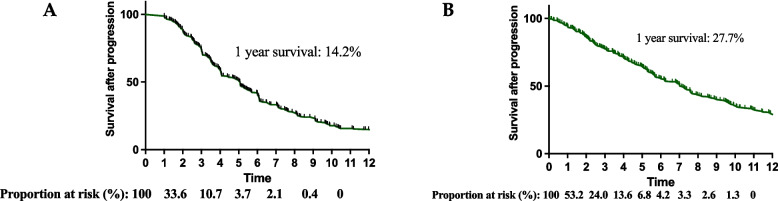
Kaplan–Meier curves of SAP of all patients in CGGA (**A**) and TCGA (**B**)

A total of 319 patients with HGGs who have been diagnosed with progressive disease were enrolled in this study including 111 anaplastic gliomas and 208 primary GBM. The clinical and molecular characteristics of recurrent patients are summarized in Table 1. The 1-year OS and PFS of recurrent patients were 68.0% (median survival 15.3 months) and 37.0% (median survival 9.7 months), respectively. The 1-year SAP was 14.2% (median survival 5.1 months) (Fig. 1A). In addition, we collected 308 patients with primary GBM from TCGA, and the characteristics were summarized in Table 2, The 1-year OS and PFS of recurrent patients were 64.9% (median survival 15.1 months) and 18.8% (median survival 5.9 months), respectively. The 1-year SAP was 27.0% (median survival 7.1 months) (Fig. 1B).

**Table 1:** Baseline clinical and tumor features of all patients in CGGA

**Characteristics**	**All patients (*n*** = **319)**
**Age**	
Mean age (range)	48.4 (17–81)
**Gender** (%)	
Male	192 (60.2%)
Female	127 (39.8%)
**Histology** (%)	
AA	36 (11.3%)
AO	17 (5.3%)
AOA	58 (18.2%)
GBM	208 (65.2%)
**Grade** (%)	
3	111 (34.8%)
4	208 (65.2%)
**TCGA subtype** (%)	
Neural	11 (13.6%)
Proneural	10 (12.3%)
Classical	20 (24.7%)
Mesenchymal	40 (49.4%)
**IDH1/2 mutation** (%)	
Yes	50 (20.2%)
No	198 (79.8%)
**MGMT promoter methylation** (%)	
Yes	130 (50.8%)
No	126 (49.2%)
**EGFR amplification** (%)	
Yes	14 (7.2%)
No	180 (92.8%)
**TP53 mutation** (%)	
Yes	21 (18.7%)
No	91 (81.3%)
**LOH1p/19q** (%)	
Yes	19 (9.8%)
No	175 (90.2%)
**Ki-67 expression** (%)	
0–**1**	82 (47.7%)
2–**3**	90 (52.3%)
**Resection** (%)	
Total	180 (62.0%)
Subtotal	112 (48.0%)
**Operation after progression**	
Yes	36 (11.3%)
No	283 (88.7%)
**TMZ after progression**	
Yes	23 (7.2%)
No	296 (92.8%)
**Therapy** (%)	
Standard RT + TMZ	105 (33.3%)
Unstandard	210 (66.7%)
**Tumor involvement** (%)	
Single lobe	181 (59.3%)
Multi-lobes	124 (40.7%)

**Table 2:** Baseline clinical and tumor features of all patients in TCGA

Characteristics	All patients (*n* = 308)
**Age**	
**Mean age (range)**	56.7 (10.9–86)
**Gender** (%)	
Male	200 (64.9%)
Female	108 (35.1%)
**Histology and grade** (%)	
GBM and 4	308 (100%)
**TCGA subtype** (%)	
Neural	43 (14.9%)
Proneural	57 (19.8%)
Classical	84 (29.2%)
Mesenchymal	104 (36.1%)
**IDH1/2 mutation** (%)	
Yes	11 (4.9%)
No	212 (95.1%)
**MGMT promoter methylation** (%)	
Yes	80 (44.4%)
No	100 (55.6%)
**Therapy** (%)	
Standard RT + TMZ	225 (73.1%)
Unstandard	83 (26.9%)

### MGMT promoter methylation is associated with prolonged SAP

In 319 samples of CGGA, clinical information, combined with IDH1 mutation, MGMT promoter methylation, TP53 mutation, EGFR amplification, 1p/19q status, Ki-67 expression, and clinical interventions were collected. Univariate Cox analysis was used to identify the associated factors with OS, PFS, and SAP (Table 3). As expected, tumor grade, IDH1 mutation, MGMT promoter methylation, and Ki-67 expression were significantly associated with OS and PFS. Tumor grade, MGMT promoter methylation, and Ki-67 expression were associated with SAP. In 308 samples of TCGA, age, MGMT promoter methylation, and therapy schedule (standard chemoradiotherapy vs. nonstandard) were associated with OS; MGMT promoter methylation and therapy schedules were associated with PFS; Age, MGMT promoter methylation, IDH1 mutation, and therapy schedules were associated with SAP (Table 4).

**Table 3:** Univariate Cox analysis of factors for OS, PFS, and SAP in CCGA

Variables	OS		PFS		SAP	
	HR (95%CI)	*p* value	HR (95%CI)	*p* value	HR (95%CI)	*p* value
**Age**						
< 60 vs. ≥ 60	1.30 (0.99–1.71)	0.06	1.27 (0.97–1.66)	0.08	1.09 (0.83–1.44)	0.53
**Gender**						
Male vs. Female	1.14 (0.90–1.43)	0.29	1.12 (0.90–1.40)	0.32	1.15 (0.91–1.45)	0.24
**Grade**						
3 vs. 4	2.06 (1.60–2.67)	0.00	1.97 (1.54–2.50)	0.00	1.42 (1.12–1.81)	0.00
**TCGA subtype**	0.94 (0.75–1.17)	0.56	1.01 (0.81–1.26)	0.92	0.85 (0.69–1.04)	0.11
**MGMT methylation**						
Yes vs. no	0.65 (0.50–0.84)	0.00	0.75 (0.58–0.96)	0.02	0.68 (0.52–0,87)	0.00
**IDH1 mutation**						
Yes vs. no	0.62 (0.45–0.86)	0.00	0.64 (0.47–0.88)	0.01	0.77 (0.56–1.06)	0.11
**TP53 mutation**						
Yes vs. no	1.16 (0.72–1.89)	0.54	1.15 (0.71–1.86)	0.58	1.04 (0.65–1.69)	0.86
**EGFR amplification**						
Yes vs. no	0.65 (0.36–1.17)	0.15	0.82 (0.48–1.42)	0.48	0.59 (0.33–1.06)	0.08
**Ki-67 expression**						
0–1 vs. 2–3	1.71 (1.25–2.34)	0.00	1.64 (1.21–2.23)	0.00	1.40 (1.03–1.92)	0.03
**LOH1p/19q**						
Yes vs. no	0.66 (0.41–1.07)	0.09	0.66 (0.41–1.06)	0.09	0.75 (0.46–1.21)	0.24
**Involved lobe**						
Single vs. multiple	1.03 (0.81–1.31)	0.79	1.00 (0.80–1.27)	0.97	1.02 (0.81–1.29)	0.87
**Resection**						
Total vs. subtotal	0.97 (0.76–1.24)	0.78	0.97 (0.77–1.24)	0.82	1.07 (0.84–1.36)	0.61
**Operation after progression**						
Yes vs. no	1.17 (0.80–1.72)	0.41	NA	NA	0.98 (0.67–1.43)	0.90
**TMZ after progression**						
Yes vs. No	1.36 (0.86–2.15)	0.19		NA	1.12 (0.71–1.76)	0.64
**Therapy**						
Standard vs. unstandard	0.86 (0.67–1.09)	0.21	0.94 (0.74–1.18)	0.59	0.86 (0.67–1.10)	0.22

**Table 4:** Univariate Cox analysis of factors for OS, PFS, and SAP in TCGA

Variables	OS		PFS		SAP	
	HR (95%CI)	*p* value	HR (95%CI)	*p* value	HR (95%CI)	*p* value
**Age**						
< 60 vs. ≥ 60	1.80 (1.40–2.31)	0.00	1.33 (1.06–1.67)	0.14	1.73 (1.35–2.23)	0.00
**Gender**						
Male vs. female	1.01 (0.79–1.31)	0.92	1.017 (0.80–1.28)	0.89	0.99 (0.77–1.28)	0.92
**TCGA subtype**	1.05 (0.93–1.18)	0.44	1.01 (0.91–1.13)	0.81	1.06 (0.94–1.19)	0.34
**MGMT** **methylation**						
Yes vs. no	0.46 (0.32–0.66)	0.00	0.71 (0.52–0.97)	0.03	0.55 (0.39–0.78)	0.00
**IDH1** **mutation**						
Yes vs. no	0.48 (0.24–0.99)	0.05	0.80 (0.42–1.52)	0.50	0.42 (0.21–0.86)	0.02
**Therapy**						
Standard vs. unstandard	0.50 (0.38–0.66)	0.00	0.60 (0.47–0.78)	0.00	0.65 (0.50–0.85)	0.00

**Fig. 2:**
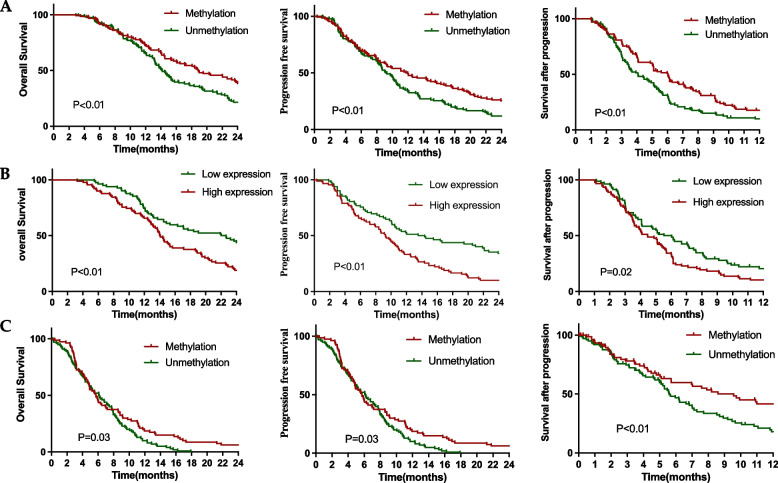
Kaplan–Meier estimates of SAP according to MGMT methylated status (**A**) and Ki-67 expression levels (**B**) in CGGA, and MGMT methylated status (**C**) in TCGA by the log-rank test

Prognostic factors associated with OS, PFS, and SAP were projected into the multivariate analysis. In CGGA, MGMT promoter methylation and Ki-67 expression were synchronously independent prognostic markers for OS, PFS, as well as SAP (Table 5, Fig. 2A, B). In the TCGA dataset, only MGMT promoter methylation is an independent prognostic marker for OS, PFS, and SAP, synchronously (Table 6, Fig. 2C). Taken together, MGMT promoter methylation is not only an independent prognostic marker for OS and PFS but also for SAP in CGGA and TCGA.

**Table 5:** Multivariable Cox regression of predictors for survival after progression in CCGA

Variables	OS		PFS		SAP	
	HR (95%CI)	*p* value	HR (95%CI)	*p* value	HR (95%CI)	*p* value
**Grade**						
3 vs. 4	1.20 (0.80–1.78)	0.39	1.18 (0.81–1.73)	0.38	1.25 (0.92–1.92)	0.13
**IDH1** **mutation**						
Yes vs. no	0.86 (0.57–1.30)	0.47	0.91 (0.60–1.36)	0.63	NA	NA
**MGMT** **methylation**						
Yes vs. no	0.52 (0.35–0.78)	0.00	0.60 (0.41–0.88)	0.01	0.68 (0.48–0.96)	0.03
**Ki-67 expression**						
0–1 vs. 2–3	1.62 (1.16–2.26)	0.01	1.51 (1.09–2.10)	0.01	1.46 (1.06–2.03)	0.02

**Table 6:** Multivariable Cox regression of predictors for survival after progression in TCGA

Variables	OS		PFS		SAP	
	HR (95%CI)	*p* value	HR (95%CI)	*p* value	HR (95%CI)	*p* value
**Age**						
< 60 vs. ≥ 60	1.28 (0.88–1.86)	0.20	1.01 (0.75–1.36)	0.94	1.47 (1.00–2.17)	0.05
**MGMT methylation**						
Yes vs. no	0.49 (0.33–0.74)	0.00	0.71 (0.52–0.97)	0.03	0.64 (0.43–0.94)	0.03
**IDH1 Mutation**						
Yes vs. no	0.45 (0.19–1.06)	0.07	NA	NA	0.32 (0.13–0.76)	0.01
**Therapy**						
Standard vs. unstandard	0.44 (0.27–0.71)	0.00	0.72 (0.49–1.06)	0.10	0.62 (0.38–1.02)	0.06

#### MGMT promoter methylation has predictive value for SAP and combined with Ki-67 expression contribute to risk-stratify

**Fig. 3:**
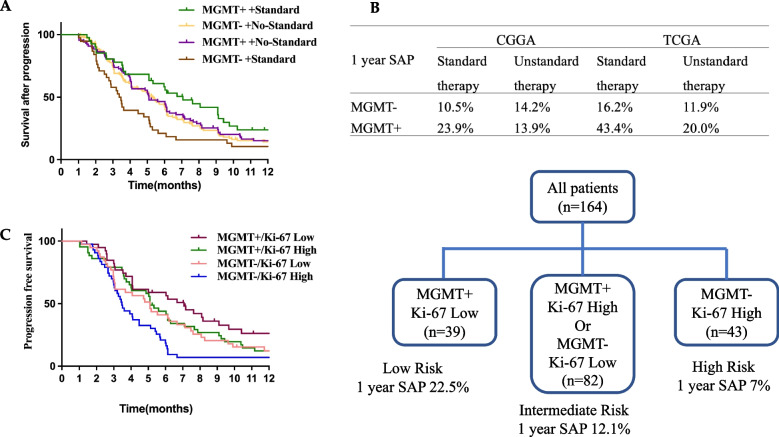
MGMT is associated with prolonged SAP. Kaplan–Meier estimates of SAP (**A**) and the 1 year-SAP (**B**) according to MGMT methylated status and treatment schedule; Kaplan–Meier estimates of SAP (**C**) and classify prognostic risk groups (**D**) according to MGMT methylated status and Ki-67 expression

Patients with HGGs containing a methylated MGMT promoter benefited from standard chemoradiotherapy (radiotherapy, and concomitant and adjuvant temozolomide). In patients treated by standard chemoradiotherapy, the median SAP in patients with MGMT promoter methylation was 7.1 months versus 3.4 months in patients with unmethylated MGMT promoter. The SAP of patients with methylated MGMT promoter was increased from 5.1 months in the nonstandard treatment group to 7.1 months in the standard group (Fig. 3A). The 1-year SAP of patients with MGMT promoter methylation is 23.9% in the standard treatment group compared with 13.9% in the nonstandard group (Fig. 3B). The result was validated in TCGA: 43.4% vs. 20.0%.

**Fig. 4:**
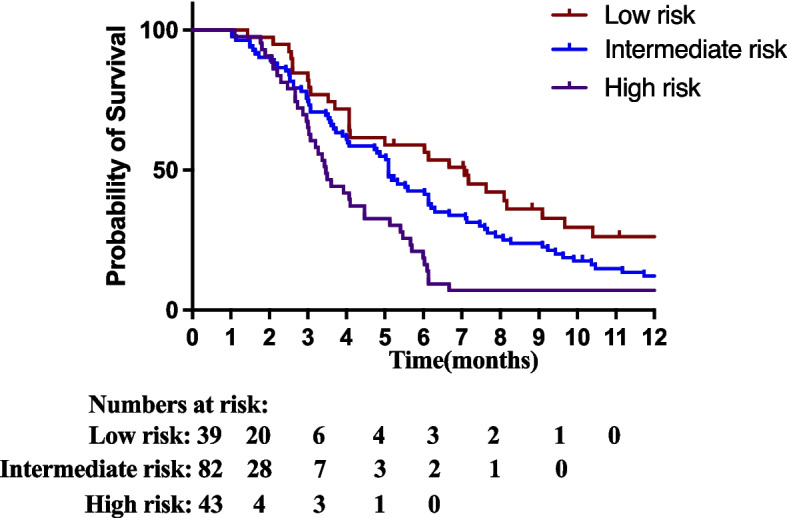
Kaplan–Meier estimates of SAP according to MGMT methylated status and Ki-67 expression levels. MGMT promoter methylation and Ki-67 low expression define low risk group; MGMT promoter unmethylation and Ki-67 high expression define a high-risk group; others define an intermediate-risk group

In our dataset, Ki-67 expression was also an independent prognostic marker for SAP. One hundred sixty-four samples available for both Ki-67 and MGMT were divided into 4 groups according to MGMT promoter methylation and Ki-67 expression. Survival analysis found that patients with MGMT promoter methylation and Ki-67 high expression and patients with MGMT promoter unmethylation and Ki-67 low expression have similar SAP (median SAP: 5.1 months vs. 5.1 months, Fig. 3C). Patients with MGMT promoter methylation and Ki-67 low expression (median SAP 7.1 months) and patients with MGMT promoter unmethylation and Ki-67 high expression (median SAP 3.5 months) have the best SAP and the worst SAP, respectively. Therefore, MGMT promoter methylation and Ki-67 low expression are defined as the low-risk group (1 year SAP 22.5%); MGMT promoter methylation and Ki-67 high expression or MGMT promoter unmethylation and Ki-67 low expression defined as intermediate risk group (1 year SAP 12.1%); MGMT promoter unmethylation and Ki-67 high expression defined as high-risk group (1 year SAP 7.0%, Fig. 3D and Fig. 4).

## Discussion

High-grade gliomas are among the most malignant tumors of the central nervous system. Tumor recurrence or progression occurs in a short time after surgery resection, mostly even during the course of chemoradiotherapy. Combined radiotherapy and temozolomide are the first-line treatment for HGGs [14]. The methylation status of the MGMT promoter is currently the most important prognostic factor and clinically relevant predictor of benefit from temozolomide chemotherapy in patients with newly diagnosed glioblastoma [6]. At present, for patients with recurrent or progressive HGGs, no definitive treatment modality has been established. However, studies have confirmed that prolonged administration of TMZ had relatively good survival despite recurrence [4, 15–19]. Gömöri et al. found methylated MGMT promoter was an early event in gliomas evolution and had been proven to be stable in tumor recurrence [20]. D.-S. Kong et al. reported MGMT promoter methylation was not an independent variable for determining the TMZ treatment outcome in 58 recurrent anaplastic gliomas [13]. However, Collins et al. found MGMT promoter methylation was an independent factor for overall survival in patients with the first recurrent high-grade gliomas [11]. In brief, the prognostic and predictive value for survival after the first progression is not clear. In this study, we collected 319 patients who were confirmed to be recurrent and progression and conducted maximum resection followed by radiotherapy and/or temozolomide chemotherapy. Of these, 256 samples with MGMT methylation status (130 methylation vs. 126 unmethylation) were analyzed for SAP. We found MGMT promoter methylation was an independent prognostic factor for SAP and patients with methylated MGMT promoter have longer SAP under standard TMZ chemoradiotherapy than unstandard treatment.

**Fig. 5:**
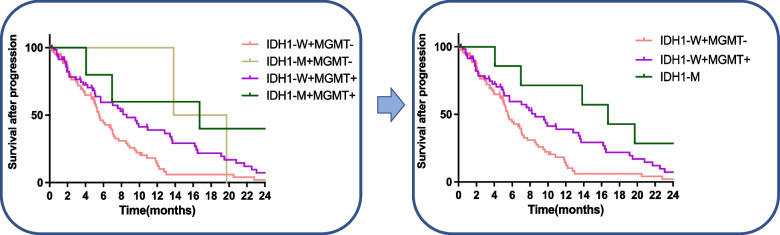
Kaplan–Meier estimates of SAP according to MGMT methylated status and IDH1 mutation status. IDH1 mutation defines the low-risk group; IDH1 wildtype and MGMT promoter methylation or unmethylation define the intermediate or high-risk group

Classifying the prognostic risk groups defined by prognostic factors may contribute to improving the accurate assessment of prognostic groups and making clinical decisions. Combined Ki-67 expression and MGMT promoter methylation define three risk groups of SAP. MGMT promoter methylation and Ki-67 low expression provided a risk reduction for SAP with standard chemoradiotherapy. More recently studies of diffuse glioma demonstrated the prognostic value of IDH1 mutation for OS and PFS [8, 9]. In patients with recurrent high-grade gliomas, IDH1/2 mutations were predictors of any type of treatment but not survival from the first progression [11]. Additional analysis found that MGMT promoter methylation combined with IDH1 mutation seemed to establish another risk model of SAP in TCGA (Fig. 5). IDH1 mutation defines the low-risk group; IDH1 wildtype with MGMT methylation or unmethylation defines the intermediated or high-risk group. Because all of the patients in TCGA were primary GBM, the validity of the conclusion remained unclear and needed further study.

In addition, the extent of resection, the first therapy schedule, surgery, or chemotherapy after the first progression were not associated with OS, PFS, and SAP in our data. The patients we collected conducted total or near-total resection (> 90%) and patients with standard chemoradiotherapy in our data were much less than that in TCGA (33.5% vs. 73.1%). Discrepancies between CGGA and TCGA might result in a discrepancy in SAP analysis. In addition, the proportion of the second operation after the first progression was only 11.3% (36/319) in CGGA and this data was not provided in TCGA. Twenty-three patients continue with TMZ treatment after the first progression in CGGA and the data is also not available in TCGA. Therefore, the treatment process after the first progression needs further study.

## Conclusions

In conclusion, MGMT promoter methylation was an independent prognostic factor for SAP and also predicted SAP of HGGs with standard TMZ chemoradiotherapy. MGMT promoter methylation, combined with Ki-67 expression or IDH1 mutation, might establish a risk model for SAP in HGG that needs further research and confirmation. This stratification in HGGs may aid in treatment strategy selection and clinical prognosis evaluation.

## Data Availability

The datasets generated and/or analyzed during the current study are available in The Chinese Glioma Genome Atlas and The Cancer Genome Atlas, http://cgga.org.cn/index.jsp and https://www.cancer.gov/ccg/research/genome-sequencing/tcga.

## Abbreviations

HGGs: High-grade gliomas
PFS: Progression-free survival
OS: Over survival
SAP: Survival After the First Progression
CGGA: The Chinese Glioma Genome Atlas
TCGA: The Cancer Genome Atlas
